# Reusable tutorials for using cloud-based computing environments for the analysis of bacterial gene expression data from bulk RNA sequencing

**DOI:** 10.1093/bib/bbae301

**Published:** 2024-07-12

**Authors:** Steven Allers, Kyle A O’Connell, Thad Carlson, David Belardo, Benjamin L King

**Affiliations:** Department of Molecular and Biomedical Sciences, University of Maine, 5735 Hitchner Hall, Orono, ME 04469, United States; Center for Information Technology, National Institutes of Health, 6555 Rock Spring Dr, Bethesda, MD 20817, United States; Health Data and AI, Deloitte Consulting LLP, 1919 N. Lynn St, Arlington, VA 22203, United States; Center for Information Technology, National Institutes of Health, 6555 Rock Spring Dr, Bethesda, MD 20817, United States; Health Data and AI, Deloitte Consulting LLP, 1919 N. Lynn St, Arlington, VA 22203, United States; Google Cloud, Google, 1900 Reston Metro Plaza, Reston, VA 20190, United States; Department of Molecular and Biomedical Sciences, University of Maine, 5735 Hitchner Hall, Orono, ME 04469, United States; Maine Institutional Development Award Network of Biomedical Research Excellence (INBRE) Data Science Core, MDI Biological Laboratory, 159 Old Bar Harbor Rd, Bar Harbor, ME 04609, United States; Graduate School of Biomedical Science and Engineering, University of Maine, 5775 Stodder Hall, Orono, ME 04469, United States

**Keywords:** gene expression, RNA sequencing, microbial genomics, analysis workflow, cloud computing, training

## Abstract

This manuscript describes the development of a resource module that is part of a learning platform named “NIGMS Sandbox for Cloud-based Learning” https://github.com/NIGMS/NIGMS-Sandbox. The overall genesis of the Sandbox is described in the editorial NIGMS Sandbox at the beginning of this Supplement. This module delivers learning materials on RNA sequencing (RNAseq) data analysis in an interactive format that uses appropriate cloud resources for data access and analyses. Biomedical research is increasingly data-driven, and dependent upon data management and analysis methods that facilitate rigorous, robust, and reproducible research. Cloud-based computing resources provide opportunities to broaden the application of bioinformatics and data science in research. Two obstacles for researchers, particularly those at small institutions, are: (i) access to bioinformatics analysis environments tailored to their research; and (ii) training in how to use Cloud-based computing resources. We developed five reusable tutorials for bulk RNAseq data analysis to address these obstacles. Using Jupyter notebooks run on the Google Cloud Platform, the tutorials guide the user through a workflow featuring an RNAseq dataset from a study of prophage altered drug resistance in *Mycobacterium chelonae*. The first tutorial uses a subset of the data so users can learn analysis steps rapidly, and the second uses the entire dataset. Next, a tutorial demonstrates how to analyze the read count data to generate lists of differentially expressed genes using R/DESeq2. Additional tutorials generate read counts using the Snakemake workflow manager and Nextflow with Google Batch. All tutorials are open-source and can be used as templates for other analysis.

## Introduction

High-throughput RNA sequencing (RNAseq) is a fundamental approach used to characterize gene expression and involves the analysis of tens of millions of sequence reads through complex analysis workflows. RNAseq can be applied to study gene expression at different scales from single cells (single-cell RNAseq), spatially distributed cells in tissue cross sections (spatial transcriptomics), to bulk tissues (bulk RNAseq). Applications of RNAseq include characterizing transcriptomes for genome annotation where gene structures are inferred by aligning reads or assembled transcripts from one or more tissues to a genome assembly. In eukaryotes, RNAseq studies have revealed extensive sets of alternatively spliced transcripts and non-coding genes. Bulk RNAseq is commonly used to characterize gene expression across multiple groups of samples containing biological replicates to determine which genes are differentially expressed. However, a major obstacle for some researchers is lack of research training in the analysis of bulk RNAseq data. The identification of differentially expressed genes requires knowledge of analysis workflows as well as how to access to substantial data storage and computing capacity depending on the size of the experiment.

**Figure 1:**
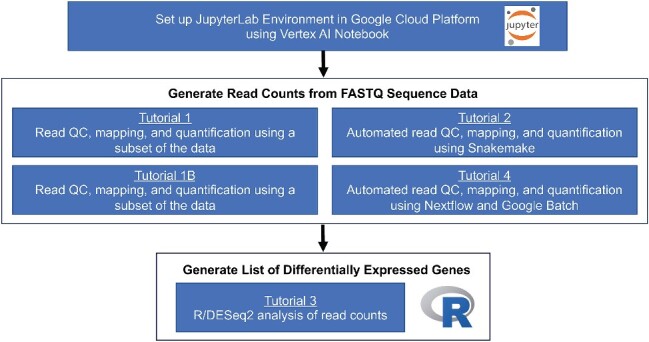
Overview of tutorials; first, instructions are given on how to use GCP’s Vertex AI Notebooks to download the tutorials from GitHub and run the tutorials; Tutorials 1, 1B, 2, and 4 analyze the FASTQ sequence files to generate the read counts per sample; those read counts are then analyzed in Tutorial 3 using R/DESeq2.

Differential gene expression analysis using bulk RNAseq has been used in a variety of experimental contexts. These range from studies of zebrafish embryonic development [1], tissue regeneration [2], to drug responses in human cells [3]. The Gene Expression Omnibus [4] had 15 465 RNA-Seq studies as of 18 July 2023 across diverse taxa. Although 12 449 of these studies were performed using human and mouse samples, there were only 507 studies performed in bacteria. In bacteria, studies include investigating biofilm formation in the human pathogen *Pseudomonas aeruginosa* [5], and gene expression in strains of *Mycobacterium chelonae* with variable drug resistance [6]. RNAseq libraries are made for each biological replicate sample where the RNA is reverse transcribed to cDNA, typically in a strand-specific manner. Since most RNA extracted from a sample are ribosomal RNAs, one option during library construction is to enrich the RNA for poly-adenylated transcripts or to deplete ribosomal RNAs. Libraries are then sequenced often using short-read sequencing platforms, such as Illumina, where reads are typically 150 bp or less. The sequence of the reads along with the quality of each base is stored as FASTQ text files. Following single-end or paired-end sequencing, reads are typically trimmed to remove sequencing adapters and filter out any low-quality sequence using tools such as Trimmomatic [7] and Trim Glore [8]. Trimmed reads are then aligned to an annotated reference genome or transcriptome using tools like Salmon [9, 10], STAR [11], RSEM [12], HISAT2 [13], or BWA [14]. The number of reads that are mapped to each transcript or gene is reported per sample using tools such as HTseq-count [15] or the “featurecounts” function in the *R* Subread package [16]. For each gene, read counts across samples are fit to a model which is then used to test whether the gene was differentially expressed between sample groups. The two most widely utilized tools for analyzing RNAseq read counts are the *R* [17] packages, edgeR [18] and DESeq2 [19]. Using these packages, the assignment of samples to sample groups is defined. Depending on the design of the experiment, sample groups are assigned to at least one experimental factor (e.g. treatment) and at least two factor levels (e.g. control and treated groups). Several diagnostic plots can be generated during the analysis of the read count data including pairwise scatterplots and principal component analysis (PCA) plots to assess variation across samples and sample groups. For each pairwise contrast of interest, these tools report a table of gene expression statistics per transcript or gene that include fold change, average level of expression, statistic (e.g. Wald test), raw *P*-value, and adjusted *P*-value. The table can then be filtered based on adjusted *P*-value or other criteria to define a set of differentially expressed transcripts or genes. Minus average (MA) and volcano plots are used to visualize the distribution of fold-change for transcripts or genes by average level of expression or adjusted *P*-values, respectively. Additional downstream analyses can be performed on sets of differentially expressed genes including clustering (e.g. heatmaps with hierarchical clustering and k-means clustering) and testing for overrepresented Gene Ontology [20] terms or Kyoto Encyclopedia of Genes and Genomes (KEGG) [21] pathways annotated to the genes.

As part of the National Institutes of General Medical Sciences (NIGMS) Sandbox Pilot Program, we developed a set of five tutorials (available at https://github.com/NIGMS/RNA-Seq-Differential-Expression-Analysis) for the analysis of bulk RNAseq data for differential gene expression studies (Fig. 1). Our materials utilize an example dataset from the previously described *M. chelonae* study [6], which used bulk RNAseq to characterize the mechanisms of antibiotic resistance in *M. chelonae* by comparing gene expression in lysogens with and without the prophage, BPs. Inclusion of the BPs prophage was shown to be associated with increased amikacin resistance and increased expression of the virulence gene, *whiB7*. We built interactive Jupyter notebooks [22] that contain the necessary command-line syntax for each step in the workflow, accompanied by documentation, from installing the software to downloading data and running each tool. As users follow instructions to reanalyze this dataset, they become familiar with command-line syntax for this specific dataset and analysis workflows. As users build skills, the materials then become a template for analyzing other RNAseq datasets. The first tutorial walks users through generating read counts for a subset of the RNAseq reads. Tutorial 1B steps users through analysis of the entire dataset and begins with downloading the reads from the NCBI Sequence Read Archive (SRA) [23]. Tutorials 2 and 4 alternatively utilize Snakemake [24] and Nextflow [25], which demonstrates workflow automation software, where all of the read counts can be generated by issuing a single command. The Nextflow tutorial uses Google Batch to issue commands. Tutorial 3 guides users through the analysis of the read counts to generate a list of differentially expressed genes using the DESeq2 package in *R*.

Our tutorials provide step-by-step instructions on how to use Google Cloud Platform (GCP) to run the analysis workflows. The primary target audience for these learning modules is undergraduate students who are interested in biomedical research at resource limited institutions. A significant obstacle for many users, especially those that are new to genomic data analysis, is having a computing platform (e.g. Linux server) with sufficient storage and compute capacity with the software installed to run analysis workflows with increasingly large and complex datasets. Cloud service providers, such as GCP, make it easy to run Jupyter notebooks with a variety of hardware and software configurations. The virtual machine created to run a particular set of Jupyter notebooks can be efficiently used such that they are stopped when not in use, or after analysis is completed, data can be migrated to the longer term Cloud Storage and the virtual machines deleted. Users also have administrative privileges on these virtual machines so that any software can be installed and sharing of files can be defined by the user. Furthermore, increasingly more genomic datasets are available on commercial Cloud service providers including sequence reads from SRA and analyzed data from The Cancer Genome Atlas via the ISB Cancer Gateway in the Cloud platform [26]. Our tutorials seamlessly combine content teaching users how to use GCP as well how to run the bioinformatic workflows. Upon completion of tutorials, learners will be able to: (i) use GCP to run bioinformatic workflows locally and via managed services; (ii) interactively download sequence data from SRA; (iii) generate read count data; (iv) generate lists of differentially expressed genes; and (v) automate workflows using Snakemake and Nextflow.

These tutorials were developed as part of the NIGMS Sandbox that was created to help train the biomedical workforce on using Cloud-based computing resources to provide broad and equitable access to high-performance computing infrastructure and increasingly large datasets [27]. There are 11 additional training modules in the NIGMS Sandbox that include the following topics: fundamentals of bioinformatics, introduction to data science for biology, analysis of biomedical data for biomarker discovery, consensus pathway analysis, DNA methylation sequencing analysis, ATAC-Seq and single cell ATAC-Seq analysis, transcriptome assembly, proteome quantification, multi-omics data integration, metagenomic analysis of biofilm-microbiome, and biomedical imaging analysis using artificial intelligence/machine learning. Usage of the NIGMS Sandbox is facilitated by other efforts at the National Institutes of Health (NIH), such as NIH Cloud Lab [28] and the NIH Science and Technology Research Infrastructure for Discovery, Experimentation, and Sustainability (STRIDES) [29] Initiative to provide access to major Cloud service providers. For example, NIH Cloud Lab currently provides NIGMS grantees and affiliates $100 in Cloud computing credits at GCP to run the NIGMS Sandbox training modules. More than 250 students, staff and faculty from these institutions had used one or more of the NIGMS Sandbox tutorials. The NIH Cloud Lab team that manages the NIGMS Sandbox tracks user feedback that will be incorporated into enhancing existing and developing new tutorials.

## Methods and implementation

We implemented the tutorials using Jupyter notebooks where documentation can be written alongside code blocks. Using GCP, we demonstrate how to use the Vertex AI Workbench to start a new virtual machine, clone the GitHub repository containing the tutorial notebooks, and walk the user through each step of the bioinformatic workflow. To run the notebooks in GCP, the following configuration must be used. The virtual server needs to be running Debian [30] version 10 with an *R* version 4.2 kernel. This environment configuration in GCP will create a server that also has JupyterLab (3.4.8) [31], Python version (3.7.12) [32], gcc (8.3.0) [33], libarchive (3.3.3-4) [34], gsutil (5.24) [35], Java version (17.0.3) [36], *R* version (4.2.3), and Perl (17.0.3) [37]. The first step in the tutorials is to install all necessary software using mamba (1.4.2) [38]. The tutorials could be run on a properly configured laptop using JupyterLab; however, the documentation we have written assumes that the user is using GCP.

**Figure 2:**
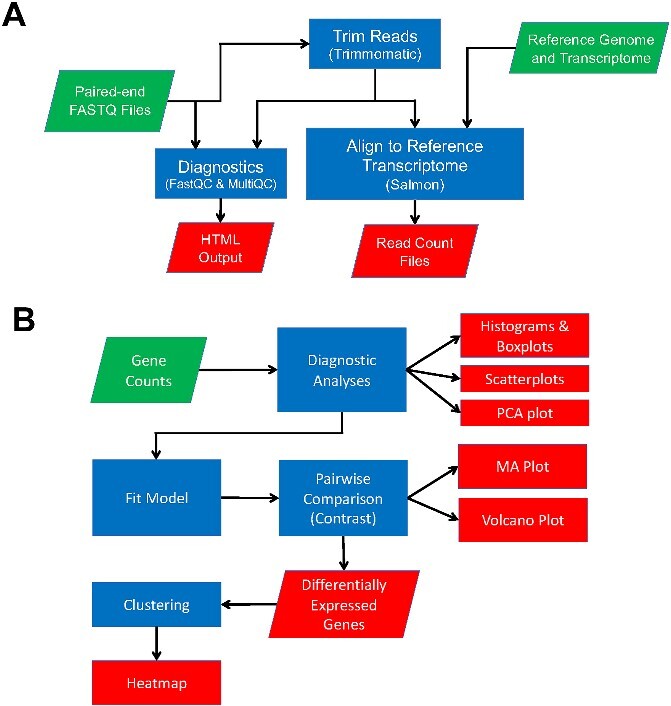
Analysis workflows; (A) read mapping workflow featured in Tutorials 1, 1B, 2, and 4. The workflow includes input files (FASTQ files, reference genome and transcriptome), analysis steps (diagnostics, read trimming, read alignment), and output files (diagnostic analysis results and read count tables); (B) analysis of read counts to test for differentially expressed genes demonstrated in Tutorial 3. Following diagnostic analyses of the input read counts, a model is fit to the data and pairwise comparisons are performed to generate lists of differentially expressed genes that are visualized using MA, volcano and heatmap plots.

A unique aspect of our tutorials is that we feature a prokaryotic RNAseq workflow, whereas other examples focus human datasets [39, 40]. The read mapping workflow (Fig. 2A) begins by copying the FASTQ files containing the reads. In Tutorial 1, the user copies down sampled data (the first 50 k reads per sample) from Google Cloud Storage. For Tutorial 1B, the user runs the sra-toolkit [41] (prefetch then fasterq-dump) to copy the full dataset from SRA. As the RNAseq libraries for each sample were sequenced in two lanes using paired-end sequencing in the experiment, the user concatenates the reads from the lane together for each sample using the UNIX utility, cat. Next, the user trims the reads using Trimmomatic (0.39), and then analyzes the trimmed reads for quality using FastQC (0.12.1) [42] and MultiQC (1.14) [43]. The user then downloads the reference genome assembly of *M. chelonae* CCUG 47445 and RefSeq [44] annotation (RefSeq accession NZ_CP007220.1) the NCBI. The user runs Salmon (1.10.2) to generate an indexed transcriptome using the assembly and the annotation. Most tutorials, or automated pipelines, demonstrate processing under ideal conditions. However, actual datasets often require special considerations. In our example analysis tutorial, we use a dataset that has high overlap between forward and reverse paired-end reads. Users are guided through how to detect and handle such eventualities. In this dataset, the reverse (R1) and forward (R2) paired-end reads were highly overlapping, so the user then only maps the reverse reads using Salmon. The output files from Salmon contain the read counts per gene for each sample as separate text files. Tutorial 3 walks the user through the read count statistical analysis workflow. The user runs the analysis using the following R packages: DESeq2 (1.38.3), ComplexHeatmap (2.14.0) [45], EnhancedVolcano (1.16.0) [46], dplyr (1.1.2) [47], pheatmap (1.0.12) [48], ggrepl (1.16.0) [49], and ggfortify (1.16.0) [50] (Fig. 2B). First, the user performs diagnostic analyses of the read counts from Salmon to examine variation among the samples using pairwise scatterplots, boxplots, and principal component analysis. After defining the experimental design, the user runs DESeq2 to determine which genes were significantly differentially expressed. The output from DESeq2 includes statistics reported per gene in a text file, an MA plot, and a volcano plot. Finally, the user generates a heatmap for the top 50 genes.

**Figure 3:**
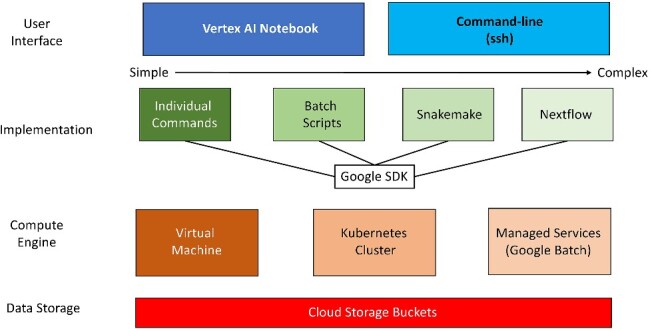
Implementation of workflow in GCP; workflows can be implemented using different combinations of user interfaces, implementation strategies, compute engines, and data storage architectures; implementation methods range from simple to more complex implementation methods that better support automation necessary to analyze large experiments; we feature tutorials that include different implementations of the same bulk RNA-seq workflow; Tutorials 1 and 3 primarily use individual commands and a virtual machine; Tutorial 2 includes the use of Snakemake to manage individual commands; Tutorial 4 uses Nextflow, an alternative to Snakemake, and directly uses the Google Batch API.

Another unique aspect of our tutorials is that we demonstrate how a workflow can be run using various computing environments within GCP, from local compute to using serverless configurations (Fig. 3). Within GCP, analysis steps can be run locally using Jupyter notebooks through the Vertex AI Workbench. Tutorials 1, 1B, and 3 use notebooks to run the separate steps. Alternatively, the commands shown in the code blocks of the notebooks could be run in the command line in the GCP terminal window or run on the command line. Tutorials 1 and 1B show how the read mapping workflow is run using separate steps, and then Tutorial 2 demonstrates how that same workflow can be automated using Snakemake. Lastly, Tutorial 4 illustrates how to run the read mapping workflow using Nextflow that can submit compute jobs to Google Batch. Both Nextflow and Google Batch can be used to parallelize workflows so that samples are analyzed in parallel rather than serially.

## Results

These freely accessible tutorials provide concrete examples of how to analyze a prokaryotic bulk RNAseq dataset using GCP. We first provide step-by-step instructions on how to use GCP to create a virtual machine to run the tutorials by launching a new notebook using GCP’s Vertex Artificial Intelligence (AI) Workbench, a JupyterLab environment. The tutorials are copied to the JupyterLab environment from the NIGMS Sandbox GitHub repository. The tutorials execute a workflow for prokaryotic gene expression analysis: (a) read preprocessing and mapping (Tutorials 1, 1B, 2, and 4), and (b) statistical analysis of read counts to test for differentially expressed genes (Tutorial 3) (Fig. 1). The first step for each of the tutorials is to install the additional analysis tools (e.g. sra-toolkit, FastQC, MultiQC, Trimmomatic, Salmon, etc.) on the virtual machine using MambaForge [51] and BioConda [52]. The read preprocessing and mapping tutorials begin by downloading the reads as FASTQ files, performing diagnostic analysis of the reads using FastQC and MultiQC, trimming reads using Trimmomatic, and read mapping and quantification using Salmon. The steps in the workflow are run by the user block-by-block so that the user can learn the syntax for running different analysis steps. However, running analysis workflows step-by-step for experiments with large numbers of samples is not efficient. One solution to automating workflows is to use Snakemake or Nextflow. Tutorial 2 implements the read mapping workflow using Snakemake, and Tutorial 4 demonstrates how to run the workflow with Nextflow using the Google Batch.

The example dataset featured in these tutorials was generated as part of a study of intrinsic antibiotic resistance in *M. chelonae*, a non-tuberculosis mycoprokaryotic pathogen [6]. The investigators discovered that a strain containing the prophage, BPs, had increased resistance over a strain without BPs. Prophages are viral genomes that are integrated into a host genome. Many genomes of mycoprokaryotic pathogens contain prophages that are hypothesized to have roles in virulence [17]. The study was designed to compare gene expression in the two *M. chelonae* strains using bulk RNAseq (Table 1). Total RNA from three biological replicate plates was extracted and ribosomal-reduced RNAseq libraries were prepared. The libraries were sequenced using paired-end 50 bp reads. In Tutorial 1, the user analyzes a subset of the reads (50 000 read pairs) for the six samples so that each step runs quickly. These truncated FASTQ files are copied from a Google storage bucket. Tutorial 1B has the user download the entire set of reads from SRA using the sra-toolkit. The *M. chelonae* CCUG 47445 assembly and associated annotation from NCBI RefSeq are used to create a transcriptome that is indexed for read mapping using Salmon. Once the reads and assembly are downloaded, the user executes each step of the workflow that results in gene-level read counts per sample.

**Table 1:** Experimental design of the RNAseq experiment (GSE164210)

Sample ID (GSM ID)	Sample description	Factor level	SRA run IDs	Number of reads	Number of trimmed reads (%)	Number of mapped reads (%)
GSM5004088	*M. chelonae* WT rep 1	WT	SRR13349122_1 SRR13349123_1	21 992 846	99.61%	95.41%
GSM5004089	*M. chelonae* WT rep 2	WT	SRR13349124_1 SRR13349125_1	21 719 959	99.61%	95.23%
GSM5004090	*M. chelonae* WT rep 3	WT	SRR13349126_1 SRR13349127_1	24 830 529	99.61%	95.90%
GSM5004091	BPs lysogen *M. chelonae* rep 1	BPs	SRR13349128_1 SRR13349129_1	25 614 180	99.61%	94.86%
GSM5004092	BPs lysogen *M. chelonae* rep 2	BPs	SRR13349130_1 SRR13349131_1	20 574 175	99.61%	95.92%
GSM5004093	BPs lysogen *M. chelonae* rep 3	BPs	SRR13349132_1 SRR13349133_1	22 945 238	99.60%	95.70%

Sample ID, description, factor level, SRA run IDs, number of reads, percent of trimmed reads, and percent of mapped reads.

**Figure 4:**
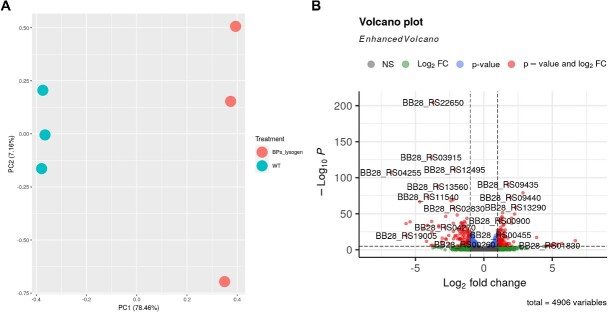
Example outputs from differential gene expression analysis of the RNAseq experiment (GSE164210); (A) PCA plot depicting clustering of sample groups and; (B) volcano plot displaying log2 fold change of gene expression (*x*-axis) and FDR (*y*-axis) of that expression; genes that are both highly differentially expressed (<0.05 FDR) as well as display high log2 fold change (>1) are displayed beyond the dotted lines.

Tutorial 3 walks the user through analyzing read counts for each of the six samples and testing for differentially expressed genes using the DESeq2 package in R. The user first installs the R packages, then they download the count data in case the user has only performed read mapping on the truncated reads in Tutorial 1A. Following best practices, the user first generates a set of diagnostic plots that include pairwise scatter plots and a principal components analysis (PCA) plot to look at the variation of gene expression among the six samples. The PCA plot showed clear separation between samples in the two sample groups (Fig. 4A). After specifying the experimental design in DESeq2 and fitting a model, the set of differentially expressed genes are generated. The user also generates a MA plot, volcano plot (Fig. 4B), and a heatmap. A tab-delimited text file containing the results of the analysis for each gene is generated containing various statistics, such as the log2 fold change, average level of gene expression, F statistic, raw *P*-value, and Benjamini–Hochberg adjusted *P*-value. A total of 1382 genes had an false discovery rate (FDR) < 0.05. As the original investigators found, the *whiB7* virulence factor was significantly upregulated in the BPs strain.

We have used these modules to train undergraduate students in the Maine INBRE Genomics Research Collaborative, and 22 students in the University of Maine Honors College Molecular Mechanisms of Human Disease course. These students used the modules as a template to write their own Jupyter notebooks to analyze different zebrafish RNAseq studies. Using GCP, it costs ~$3.00 to run the modules assuming an “n1-standard-8” (8 virtual CPUs with 32 GB of RAM) machine with 100 GB of disk storage.

## Discussion

The tutorials we developed fill an important need to train researchers and students in biomedical data science as they demonstrate how to both create a computing environment and use the tools to analyze a dataset. Although many analysis tools (e.g. sra-toolkit) may have helpful documentation, the authors of those tutorials assume that you have a server capable of storing large volumes of data files or running large compute jobs, and experience using high-performance computing environments. Here, we step the user through the process of creating a virtual machine, installing all required software, downloading data, and executing the analysis workflow. These materials are open source; they are free and publicly available to the research community.

**Figure 5:**
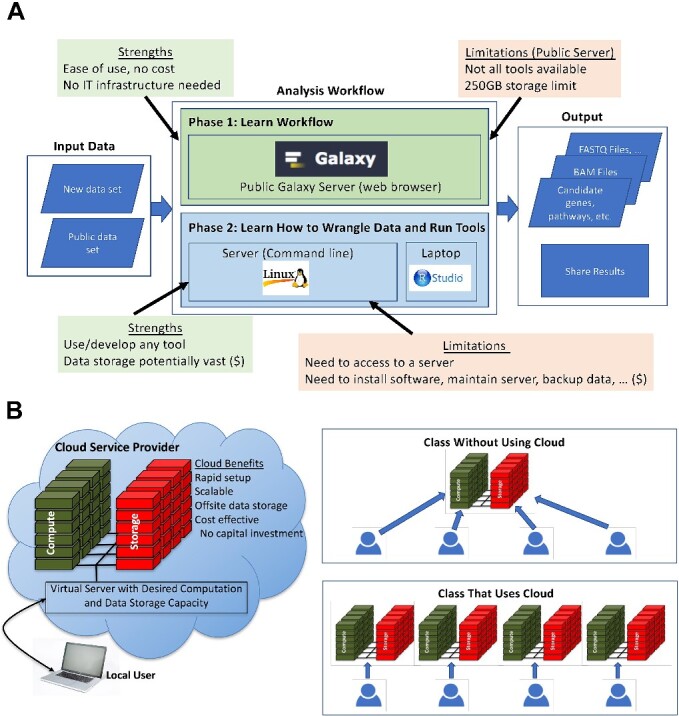
Training paradigms for high-throughput sequencing analysis workflows; (A) advantages and disadvantages of computing environments used for group training; analysis platforms such as Galaxy are free-to-use and easy to set up; but public servers contain data-limit caps, as well as limitations in terms of the types and versions of software available; in contrast, directly using tools on a server allows a greater level of customization in program usage and installation, as well as data storage; however, these include server maintenance costs, and software and workflows are less easy to set up and run; (B) cloud computing offers a compromise between such paradigms; virtual machines can offer high customization and high scalability at affordable pricing, both for processing and data-storage; machine set up is relatively simple, and installation and usage of programs can be made easy to use using shareable notebook tutorials; additionally, classes that use cloud computing have students all use the same resources, providing equity in computing resources each student can access, and removes the complications installing software on individual student personal computers; finally, all this is achieved without needing separately managed local or remote high-performance computing clusters.

There are several computing platforms that are commonly used in training individuals to analyze large datasets through workshops and courses (Fig. 5A). Individuals learning these skills must have access to a computing system with sufficient resources to store and run analyses, which can be substantial. Some courses use the public Galaxy [18] server which has several advantages and some limitations. The web-based Galaxy interface makes it easy for users to run analysis steps on large datasets, maintain and share histories, and automate workflows. This powerful platform allows users to run tools and workflows, but currently has a data storage limit of 250 GB per users on the freely accessible public site. One advantage of Galaxy is that the user can focus on learning about the tools and workflow with no logistical or financial barriers. However, users don’t get to see the complexities of how to download the data, manage files, install software, run each step of the workflow, and learn command-line syntax. Understanding these complexities is important when the characteristics of a particular dataset do not meet assumptions made in highly automated analysis pipelines. An alternative to using Galaxy for training courses is to use a centralized server or cluster that everyone uses to run their analyses. This provides the opportunity for students to learn all the technical details of using a server, such as command-line syntax. One downside of using a centralized server or cluster is that they are typically managed by someone else. Depending on how the server is managed, the user may have storage or other limitations, including permission to install software on the server. Foremost among these is that the user may not be able to access the server after the workshop or course is complete. The individual is then challenged to create a similar computing environment at their home institution so that they can perform the analyses that they had just learned at the workshop. In some cases, it may not be possible for them to do that because they don’t have the technical expertise or other institutional barriers. Traditionally, an individual would need to buy a server, house the server in an appropriate environment, and have the technical expertise to set up and administer the server. Cloud computing services provide a solution to these problems [53–55]. Our tutorials show users how to create the necessary compute environment on their own. Analysis run times can be shortened by having each student use their own virtual server rather than sharing one single centralized server (Fig. 5B). Lastly, our tutorials serve as templates for the users to re-use to analyze other datasets and/or change the analysis tools used in the workflows.

Although Cloud services are an efficient solution to creating these computing environments, there can be institutional obstacles to using these services. Users need to sign up for an account with commercial service providers. While this may be easy for individual to set up independent of an institution, there may be substantial barriers at the institutional level. An institution needs to have agreements in place with the service provider before the user can obtain an account. While large academic institutions are likely to already have these agreements in place, small institutions may not. Once agreements are in place, users then need to work out a solution for billing to occur seamlessly which can include working with a reseller. Once an institution has been onboarded, NIH-funded investigators at that institution can leverage discounted rates on major commercial service providers through the STRIDES. STRIDES provides support to investigators in learning the basics of Cloud computing and navigating through the process of onboarding institutions.

A major focus of our pilot project with Maine INBRE, Google, Deloitte, and NIGMS was to explore how researchers from small institutions could leverage Cloud computing to advance biomedical data science training. Since we recognized the potential institutional barriers of onboarding small institutions, we explored the use of Binder [56] to run Jupyter notebooks on a virtual machine without an account using GCP. Binder is a web service that allows users to clone notebooks and then run them using JupyterHub and Kubernetes [57]. Binder is potentially powerful because it removes all barriers to researchers and students to learn biomedical data science workflows, including our tutorials. Although we did implement a working system, concerns around data security and cost posed substantial challenges. Ongoing efforts, such as the NIH Cloud Lab [28], provide resources for Cloud computing research and training environments.

**Figure 6:**
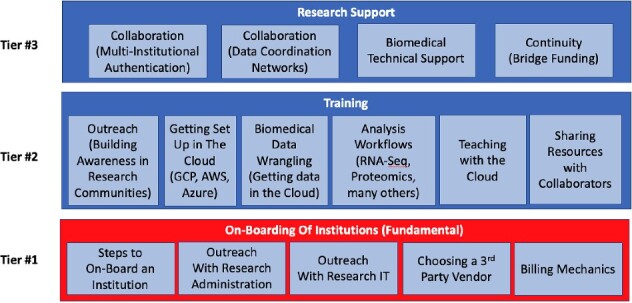
Multi-tiered approach to facilitate the application of Cloud-based biomedical data science; there are three tiers of training and support needed to successfully apply Cloud computing in biomedical data science research and training; Tier #1 includes on-boarding institutions so that license agreements, accounts, and billing mechanics are set up with the Cloud service provider; Tier #2 is an array of training activities to cover a variety of topics where participants receive hands-on experience in a variety of data science use cases; Tier #3 is a layer of support needed to help researchers successfully utilize Cloud-based biomedical data science in their research.

**Table 2:** Major decision points for re-using the workflow to generate read counts from FASTQ sequence data

Generate read counts from FASTQ sequence data (Tutorials 1, 1B)	
*Upload FASTQ files*	
Retrieve data from SRA for other experiments	Use sra-toolkit with syntax similar to what is shown in Step 3 with the SRR identifiers of interest.
Local files	Upload files to a Storage Bucket using the Google Cloud console and use gsutil to copy the files onto the server using syntax similar to what is shown in Step 3.
*Read trimming and QC*	
Single-end or paired-end reads	If single-end reads were generated instead of paired-end reads, run trimmomatic using the “PE” instead of “SE” argument, specify one input FASTQ file, and one output file (see Step 6). Run FastQC on each trimmed FASTQ file (see Step 7).
Sequencing adapters	If adapters are not represented in the TruSeq3-PE.fa file, then create a FASTA file containing the adapters and upload it to a Storage Bucket using the Google Cloud console and use gsutil to copy the files onto the server using syntax similar to what is shown in Step 5.
*Reference genome and annotation*	
Genome assembly (FASTA file), and annotation (GTF file)	For genomes available in NCBI RefSeq, use syntax similar to Step 4 to download the assembly and annotation using esearch. Alternatively, assemblies may also be downloaded from Ensembl, Ensembl Metazoa, Ensembl Plants, Ensembl Bacteria, Ensembl Fungi, or Ensembl Protists using wget and specifying the URL to the assembly file like this (below is for *M. chelonae strain* B17_S14 assembly): wget https://ftp.ensemblgenomes.ebi.ac.uk/pub/release-59/bacteria/fasta/bacteria_35_collection/mycobacteroides_chelonae_gca_001853775/dna/Mycobacteroides_chelonae_gca_001853775.ASM185377v1.dna_sm.toplevel.fa.gz The corresponding Ensembl GTF file may be download using wget: wget https://ftp.ensemblgenomes.ebi.ac.uk/pub/release-59/bacteria//gtf/bacteria_35_collection/mycobacteroides_chelonae_gca_001853775/Mycobacteroides_chelonae_gca_001853775.ASM185377v1.59.gtf.gz
*Read alignment method*	
Align to trasnscriptome or whole genome	Reads may be aligned to the transcriptome or whole genome. Alignment to a transcriptome relies on having accurate genome annotation. The tutorials use Salmon to conduct those alignments (see Steps 9 and 10), but other tools are available (e.g. RSEM). Reads aligned to the whole genome can be used to annotate exons, transcripts, and genes. For analysis of eukaryotic RNA-Seq data, a splice-aware aligner that allows for gapped alignments of reads must be used (e.g. STAR, HISAT2). Whichever aligner is used will require the generation of index files for the genome assembly (see Step 9).
Single-end or paired-end reads	For alignment of paired-end reads using Salmon, the two trimmed FASTQ files (R1 and R2) are specified using the −1 and − 2 arguments, respectively, instead of the −r argument shown in Step 10.
Strand-specific libraries	Most RNA-Seq libraries are “reverse stranded” where the R1 reads were derived from the reverse strand and aligners will take that into account. For Salmon, the library type argument should be used as “-l SR” for single-end reads (as shown in Step 10), and “-l ISR” for paired-end reads.
Evaluate mapping rates	Regardless of read alignment strategy, evaluate the percent of reads that align to the reference. A low mapping rate may indicate a problem in the analysis or potentially sample or library preparation.
*Read counting*	
Transcript-level or gene-level	Depending on the tool used, read counts may be generated per transcript or per gene (across all exons). Salmon can be used to both align the reads and generate read counts per transcript. Other tools, such as RSEM, generate both transcript and gene-level output files. HTseq-count is best used to generate read counts per gene as it considers reads that map to exons shared among transcripts as ambiguously mapped reads.
Strand-specific libraries	Like read aligners, read counting tools will take strand into account when using the correct command-line arguments. Most RNA-Seq libraries are “reverse stranded” where the R1 reads were derived from the reverse strand.
Multimapping reads	Some reads may align to multiple transcripts or genes and read counting tools can be configured to exclude them or take them into account. Salmon uses a file of “decoy” sequences to avoid spurious mappings [10]. RSEM uses expectation maximization to estimate read abundance.
Evaluate distribution of read counts	Regardless of read counting strategy, evaluate the distribution of reads across transcripts/genes. Low read counts may indicate a problem in the analysis if the mapping rates were high.

Re-using the workflow in Tutorials 1 and 1B for other datasets will require modifying the FASTQ files to upload, how read trimming and QC analysis is performed, the genome assembly and annotation used, potentially the read alignment and read counting methods used.

**Table 3:** Major decision points for re-using the workflow for generating lists of differentially expressed genes.

Generate lists of differentially expressed genes (Tutorial 3)	
*Experimental design*	
Define how samples belong to sample groups	The experimental design matrix provides a mapping of how each sample maps to sample groups (see Step 4). Here, we have a single treatment factor and two factor levels. The “samples” data frame would need to be changed to represent other experiments.
*Sample QC and read preprocessing*	
Remove lowly expressed genes	Since not all genes are expressed in a given sample, it is useful to exclude lowly expressed genes from the analysis. Step 5 removes all genes that have < 10 reads across all samples. This is an arbitrary threshold that may need to be adjusted depending on the number of samples in the experiment (e.g. increased with more samples).
Pairwise scatterplots	Evaluate the pairwise scatterplots to look for bias among samples (Step 6). Ideally, there would be lower within group variation than between groups.
PCA plot	Evaluate the PCA plot to examine the overall variation between samples (Step 7). Ideally, biological replicate sample would be clustered together and separate from other sample groups.
*Pairwise comparisons and reporting results*	
Pairwise comparisons	Step 8 performs a pairwise comparison between the two sample groups. With other experiments there may be more than one possible pairwise comparison. Results are annotated and written to tab-delimited output files in Step 9. Volcano and MA plots are generated in Step 10. The code in Steps 8–10 could be re-used for additional comparisons.
Annotating results	Step 9 annotates the output of the pairwise comparison with gene symbol, chromosome coordinates, and other information. When analyzing other genomes, an annotation file needs to be created and then uploaded to a Storage Bucket and then copied to the server using gsutils. If using Ensembl annotation, BioMart is a convenient tool that can be used to generate the annotation file. The annotation file must use the same unique identifier for the transcript/gene used when generating the read counts.

Re-using the workflow in Tutorial 3 for other datasets will require modifying the experimental design, carefully examining sample QC diagnostic plots, and defining the pairwise comparisons used to generate the lists of differentially expressed genes.

Our pilot project served as an important example of how a collaboration between a federal agency, academic researchers, and the private sector can work together to advance biomedical data science training. Without partnerships it would not have been feasible to effectively coordinate the expertise and resources necessary to implement and evaluate these technologies. During our collaboration, we developed a multi-tiered approach to supporting data science research training (Fig. 6). The first tier is to support onboarding of institutions so that researchers and students can gain access to commercial Cloud computing resources, such as those with partnerships with STRIDES. Maine INBRE is a network of 16 institutions across the state and each of them will have to navigate through the process of obtaining the necessary agreements. The second tier is to provide hands-on research training in learning everything from the mechanics of logging onto a Cloud provider to running analysis workflows, to sharing data with collaborators. The third tier is supporting researchers who actively use Cloud computing resources.

These tutorials provide biomedical data science training by illustrating a bulk RNAseq workflow using GCP. We structured the tutorials so that different data or different tools could be easily customized to perform analysis of new datasets or the re-analysis of existing datasets with minor modifications (see Tables 2 and 3). It is our goal to illustrate how any user can use Cloud services to access powerful state-of-the art computing resources that may not be accessible at their own institution. Furthermore, we illustrate how Jupyter notebooks can be used to implement best-practices for analyses and make analysis workflows reproducible for other researchers. Following the completion of this pilot project along with a similar project with the IDeA National Resource for Quantitative Proteomics at the University of Arkansas for Medical Sciences, NIGMS funded 10 additional projects to develop training tutorials for a variety of analysis workflows. The researchers who led the development of the NIGMS Sandbox tutorials were from NIGMS-funded programs that focus on workforce development, such as INBRE programs that include networks of primarily undergraduate and research institutions. The tutorials in the NIGMS Sandbox provide a broad set of analysis workflows that can be built upon in future iterations and serve as a valuable and lasting resource for Cloud-based biomedical training and research.

**Key Points**

- We present a set of tutorials for the analysis of bulk RNAseq data from prokaryotic organisms. The workflow begins with the FASTQ sequence data files and includes read preprocessing, diagnostics, mapping, quantification, and statistical analysis to test for differentially expressed genes. Tutorials are provided as Jupyter notebooks that can be run on various platforms.
- Cloud services can be used to create computing environments for biomedical data science research and training. These services can help overcome obstacles in accessing computing environments with the necessary software, compute, and data storage capacity for genomics research.
- Tutorial documentation includes information on how to use the GCP to run the bulk RNA sequence data analysis workflow.
- The tutorials demonstrate how to run the same read preprocessing, diagnostics, mapping, and quantification analyses using different workflow management tools. These tools are necessary to automate analyses for experiments with large sample sizes. These include Snakemake, Nextflow, and Google Batch.

## Data Availability

All code is available at GitHub (https://github.com/NIGMS/RNA-Seq-Differential-Expression-Analysis).
